# Supplementary material for: Selector: A General Python Library for Diverse Subset Selection
Source: bioRxiv. 2025 Nov 22:2025.11.21.689756. Preprint. [Version 1] doi: 10.1101/2025.11.21.689756 (PMC12667815; doi:10.1101/2025.11.21.689756)
Supplement: Supplement 1 [file media-1.pdf]

Supporting Information

Selector: A General Python Library for Diverse Subset Selection

Fanwang Meng,[*,†,‡] Marco Martínez González,[‡] Valerii Chuiko,[‡] Alireza Tehrani,[†] Abdul Rahman Al Nabulsi,[‡] Abigail Broscius,[†] Hasan Khaleel,[‡] Kenneth López-Pérez,[¶] Ramón Alain Miranda-Quintana,[*,¶] Paul W. Ayers,[*,‡] and Farnaz Heidar-Zadeh[*,†]

†*Department of Chemistry, Queen's University, 90 Bader Lane, Kingston, Ontario, Canada, K7L 3N6*

‡*Department of Chemistry and Chemical Biology, McMaster University, 1280 Main Street West, Hamilton, Ontario, Canada, L8S 4L8*

¶*Department of Chemistry and Quantum Theory Project, University of Florida, Gainesville, Florida, USA, 32603*

E-mail: fanwang.meng@queensu.ca; quintana@chem.ufl.edu; ayers@mcmaster.ca; farnaz.heidarzadeh@queensu.ca

List of Tables

SI 1 SMILES strings for the manually-curated biased chemical library with 145 compounds, which contains 98 alkanes, 19 alcohols, 10 aldehydes, 9 ketones, 7 benzene derivatives and 2 cycloalkanes. 10

List of Figures

SI 1 Comparison of distance-based (A) and partition-based (B) selectors for selecting 50 samples from a set of size 500 in 2D dimensions. For (B), The equis-(in)dependent and equif-(in)dependent labels refer to the (in)dependent variants of the equisize and equifrequent grid partitioning method. (A) shows that MaxSum algorithm selects samples from the peripheral space occupied by the data points. When the number of selected samples s is fewer than the number of dimensions d, this ensures that each dimension is adequately represented in the chosen subset. 9

SI 2 Comparing NSimilarity based selection methods. 17

SI 3 Running time comparisons of different subset selection algorithms. The 1024-bit ECFP4 fingerprints were computed with `RDKit` for the 14k logP dataset from EPA public data[1]. The `MaxMinPicker` method in `RDKit` was as the base line. It is noticed OptiSim are slower because the setup of searching the optimal radius in `Selector` in order to get the required number of selected data points. 17

SI 4 The four-well potential with four minima: $V_A(-1.17, 1.56) = 2.84$, $V_B(-1.29, -1.53) = 2.28$, $V_C(1.29, -1.65) = 2.01$, and $V_D(1.4, 1.78) = 1.77$ kcal/mol.[2] 18

SI 5 Selecting subsets (colored with cyan circles) of four-well potential with MaxSum (1st and 3rd columns) and OptiSim (2nd and 4th columns) methods from a dataset of 10,000 points (colored with black stars) generated with Monte Carlo sampling using values of k=10 (two left columns) and k=2 (two right columns). 18
SI 6 Selecting subsets (colored with cyan circles) of four-well potential with Grid Partition Equifrequent Dependent (1st and 3rd columns) and Independent (2nd and 4th columns) methods from a dataset of 10,000 points (colored with black stars) generated with Monte Carlo sampling using values of k=10 (two left columns) and k=2 (two right columns). For the Grid Partition, 5 bins are used along each access. 19
SI 7 Selecting subsets (colored with cyan circles) of four-well potential with Grid Partition Equisize Dependent (1st and 3rd columns) and Independent (2nd and 4th columns) methods from a dataset of 10,000 points (colored with black stars) generated with Monte Carlo sampling using values of k=10 (two left columns) and k=2 (two right columns). For the Grid Partition, 5 bins are used along each access. 19
SI 8 Diversity selection measurements of ChEMBL204 Ki subset ($n_{total} = 2754$) selection with different selection methods. (A) Log-determinant of the selected subset. (B) Wasserstein distance to uniform distribution (WDUD) of the selected subset. (C) Shannon entropy of the selected subset. (D) Gini coefficient of the selected subset. Based on the WDUD, MaxSum and NSimilarity perform the best across the different fractions. Grid partitioning has the lowest diversity when less than 30% of the data is selected, but for larger fractions of the data DISE performs worst. The Gini coefficient, MaxSum and NSimilarity consistently yield the most diverse sets and DISE consistently gives the least diverse set. 20

SI 9 Diversity selection measurements of ChEMBL1871 Ki subset ($n_{total} = 659$) selection with different selection methods. (A) Log-determinant of the selected subset. (B) Wasserstein distance to uniform distribution (WDUD) of the selected subset. (C) Shannon entropy of the selected subset. (D) Gini coefficient of the selected subset. 21
SI 10 Diversity selection measurements of ChEMBL2034 Ki subset ($n_{total} = 750$) selection with different selection methods. (A) Log-determinant of the selected subset. (B) Wasserstein distance to uniform distribution (WDUD) of the selected subset. (C) Shannon entropy of the selected subset. (D) Gini coefficient of the selected subset. 22
SI 11 Diversity selection measurements of ChEMBL2047 EC50 subset ($n_{total} = 631$) selection with different selection methods. (A) Log-determinant of the selected subset. (B) Wasserstein distance to uniform distribution (WDUD) of the selected subset. (C) Shannon entropy of the selected subset. (D) Gini coefficient of the selected subset. 23
SI 12 Diversity selection measurements of ChEMBL2147 Ki subset ($n_{total} = 1456$) selection with different selection methods. (A) Log-determinant of the selected subset. (B) Wasserstein distance to uniform distribution (WDUD) of the selected subset. (C) Shannon entropy of the selected subset. (D) Gini coefficient of the selected subset. 24
SI 13 Diversity selection measurements of ChEMBL214 Ki subset ($n_{total} = 3317$) selection with different selection methods. (A) Log-determinant of the selected subset. (B) Wasserstein distance to uniform distribution (WDUD) of the selected subset. (C) Shannon entropy of the selected subset. (D) Gini coefficient of the selected subset. 25

SI 14 Diversity selection measurements of ChEMBL218 EC50 subset ($n_{total} = 1031$) selection with different selection methods. (A) Log-determinant of the selected subset. (B) Wasserstein distance to uniform distribution (WDUD) of the selected subset. (C) Shannon entropy of the selected subset. (D) Gini coefficient of the selected subset. 26
SI 15 Diversity selection measurements of ChEMBL219 Ki subset ($n_{total} = 1859$) selection with different selection methods. (A) Log-determinant of the selected subset. (B) Wasserstein distance to uniform distribution (WDUD) of the selected subset. (C) Shannon entropy of the selected subset. (D) Gini coefficient of the selected subset. 27
SI 16 Diversity selection measurements of ChEMBL228 Ki subset ($n_{total} = 1704$) selection with different selection methods. 28
SI 17 Diversity selection measurements of ChEMBL231 Ki subset ($n_{total} = 973$) selection with different selection methods. (A) Log-determinant of the selected subset. (B) Wasserstein distance to uniform distribution (WDUD) of the selected subset. (C) Shannon entropy of the selected subset. (D) Gini coefficient of the selected subset. 29
SI 18 Diversity selection measurements of ChEMBL233 Ki subset ($n_{total} = 3142$) selection with different selection methods. (A) Log-determinant of the selected subset. (B) Wasserstein distance to uniform distribution (WDUD) of the selected subset. (C) Shannon entropy of the selected subset. (D) Gini coefficient of the selected subset. 30
SI 19 Diversity selection measurements of ChEMBL234 Ki subset ($n_{total} = 3657$) selection with different selection methods. (A) Log-determinant of the selected subset. (B) Wasserstein distance to uniform distribution (WDUD) of the selected subset. (C) Shannon entropy of the selected subset. (D) Gini coefficient of the selected subset. 31

SI 20 Diversity selection measurements of ChEMBL235 EC50 subset ($n_{total} = 2349$) selection with different selection methods. (A) Log-determinant of the selected subset. (B) Wasserstein distance to uniform distribution (WDUD) of the selected subset. (C) Shannon entropy of the selected subset. (D) Gini coefficient of the selected subset. 32
SI 21 Diversity selection measurements of ChEMBL236 Ki subset ($n_{total} = 2598$) selection with different selection methods. (A) Log-determinant of the selected subset. (B) Wasserstein distance to uniform distribution (WDUD) of the selected subset. (C) Shannon entropy of the selected subset. (D) Gini coefficient of the selected subset. 33
SI 22 Diversity selection measurements of ChEMBL237 EC50 subset ($n_{total} = 955$) selection with different selection methods. 34
SI 23 Diversity selection measurements of ChEMBL237 Ki subset ($n_{total} = 2602$) selection with different selection methods. (A) Log-determinant of the selected subset. (B) Wasserstein distance to uniform distribution (WDUD) of the selected subset. (C) Shannon entropy of the selected subset. (D) Gini coefficient of the selected subset. 35
SI 24 Diversity selection measurements of ChEMBL238 Ki subset ($n_{total} = 1052$) selection with different selection methods. (A) Log-determinant of the selected subset. (B) Wasserstein distance to uniform distribution (WDUD) of the selected subset. (C) Shannon entropy of the selected subset. (D) Gini coefficient of the selected subset. 36
SI 25 Diversity selection measurements of ChEMBL239 EC50 subset ($n_{total} = 1721$) selection with different selection methods. (A) Log-determinant of the selected subset. (B) Wasserstein distance to uniform distribution (WDUD) of the selected subset. (C) Shannon entropy of the selected subset. (D) Gini coefficient of the selected subset. 37

SI 26 Diversity selection measurements of ChEMBL244 Ki subset ($n_{total} = 3097$) selection with different selection methods. (A) Log-determinant of the selected subset. (B) Wasserstein distance to uniform distribution (WDUD) of the selected subset. (C) Shannon entropy of the selected subset. (D) Gini coefficient of the selected subset. 38
SI 27 Diversity selection measurements of ChEMBL264 Ki subset ($n_{total} = 2862$) selection with different selection methods. (A) Log-determinant of the selected subset. (B) Wasserstein distance to uniform distribution (WDUD) of the selected subset. (C) Shannon entropy of the selected subset. (D) Gini coefficient of the selected subset. 39
SI 28 Diversity selection measurements of ChEMBL2835 Ki subset ($n_{total} = 615$) selection with different selection methods. (A) Log-determinant of the selected subset. (B) Wasserstein distance to uniform distribution (WDUD) of the selected subset. (C) Shannon entropy of the selected subset. (D) Gini coefficient of the selected subset. 40
SI 29 Diversity selection measurements of ChEMBL287 Ki subset ($n_{total} = 1328$) selection with different selection methods. (A) Log-determinant of the selected subset. (B) Wasserstein distance to uniform distribution (WDUD) of the selected subset. (C) Shannon entropy of the selected subset. (D) Gini coefficient of the selected subset. 41
SI 30 Diversity selection measurements of ChEMBL2971 Ki subset ($n_{total} = 976$) selection with different selection methods. (A) Log-determinant of the selected subset. (B) Wasserstein distance to uniform distribution (WDUD) of the selected subset. (C) Shannon entropy of the selected subset. (D) Gini coefficient of the selected subset. 42

SI 31 Diversity selection measurements of ChEMBL3979 EC50 subset (n_{total} = 1125) selection with different selection methods. (A) Log-determinant of the selected subset. (B) Wasserstein distance to uniform distribution (WDUD) of the selected subset. (C) Shannon entropy of the selected subset. (D) Gini coefficient of the selected subset. 43
SI 32 Diversity selection measurements of ChEMBL4005 Ki subset (n_{total} = 960) selection with different selection methods. (A) Log-determinant of the selected subset. (B) Wasserstein distance to uniform distribution (WDUD) of the selected subset. (C) Shannon entropy of the selected subset. (D) Gini coefficient of the selected subset. 44
SI 33 Diversity selection measurements of ChEMBL4616 EC50 subset (n_{total} = 682) selection with different selection methods. (A) Log-determinant of the selected subset. (B) Wasserstein distance to uniform distribution (WDUD) of the selected subset. (C) Shannon entropy of the selected subset. (D) Gini coefficient of the selected subset. 45
SI 34 Diversity selection measurements of ChEMBL4792 Ki subset (n_{total} = 1471) selection with different selection methods. (A) Log-determinant of the selected subset. (B) Wasserstein distance to uniform distribution (WDUD) of the selected subset. (C) Shannon entropy of the selected subset. (D) Gini coefficient of the selected subset. 46

SI 1 Four-Well Potential Construction

We used the four-well potential, plotted in Figure SI 4, which is described by:[2,3]

$$V(q_1,q_2) = V_0 + \sum_{i=0}^{4} a_i \exp\left[-\sigma_i^{q_1}(q_1-\alpha_i)^2 - \sigma_i^{q_2}(q_2-\beta_i)^2\right] \tag{1}$$

where $V_0 = 5.0$ kcal/mol, vector $a = [0.6, 3.0, 1.5, 3.2, 2.0]^T$ in kcal/mol, vector $\sigma^{q_1} = [1.0, 0.3, 1.0, 0.4, 1.0]^T$ in Å^{-2}, vector $\sigma^{q_2} = [1.0, 0.4, 1.0, 1.0, 0.1]^T$ in Å^{-2}, vector $\alpha = [0.1, 1.3, -1.5, 1.4, -1.3]^T$ in Å, and vector $\beta = [0.1, -1.6, -1.7, 1.8, 1.23]^T$ in Å. The four local minima labeled in Figure SI 4 correspond to $V_A(-1.17, 1.56) = 2.8450$, $V_B(-1.29, -1.53) = 2.2779$, $V_C(1.29, -1.65) = 2.0082$, and $V_D(1.4, 1.78) = 1.7755$ kcal/mol.

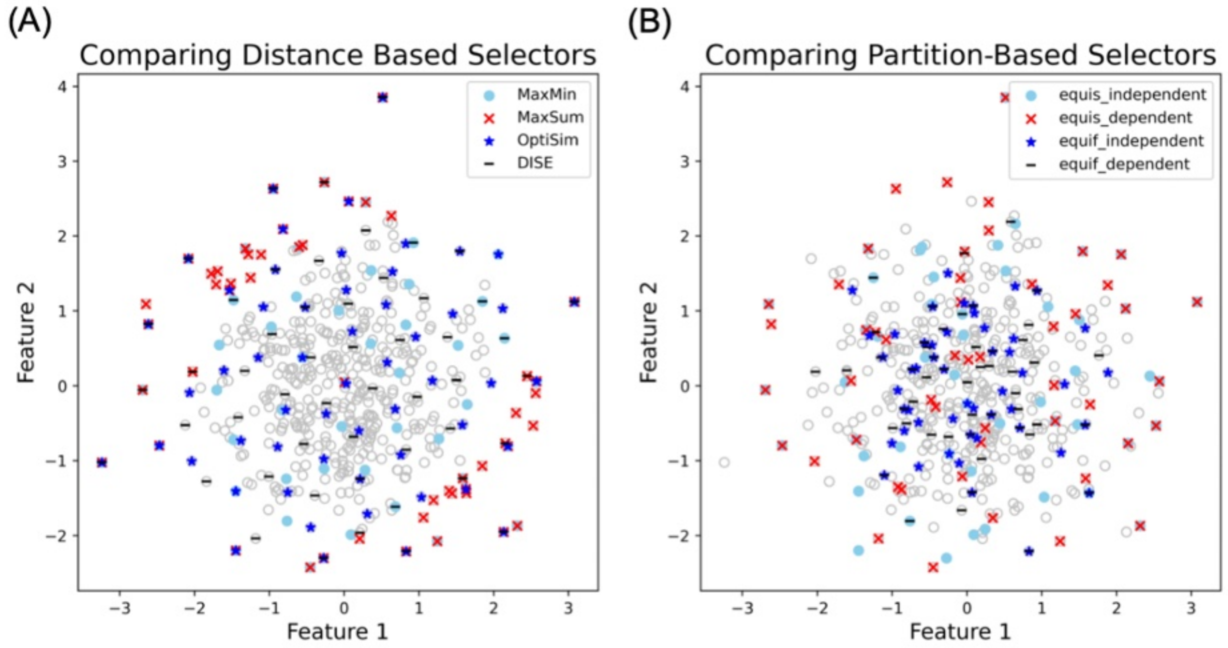

Fig. SI 1 Comparison of distance-based (A) and partition-based (B) selectors for selecting 50 samples from a set of size 500 in 2D dimensions. For (B), The equis-(in)dependent and equif-(in)dependent labels refer to the (in)dependent variants of the equisize and equifrequent grid partitioning method. (A) shows that MaxSum algorithm selects samples from the peripheral space occupied by the data points. When the number of selected samples s is fewer than the number of dimensions d, this ensures that each dimension is adequately represented in the chosen subset.

Table SI 1 SMILES strings for the manually-curated biased chemical library with 145 compounds, which contains 98 alkanes, 19 alcohols, 10 aldehydes, 9 ketones, 7 benzene derivatives and 2 cycloalkanes.

NO.	SMILES	Category
1	ketone	CCCCCC(=O)CC
2	aldehyde	CCC(C)C=O
3	benzene derivative	Nc1ccccc1
4	ketone	CCCC(C)=O
5	alkane	CCC(CC)(C(C)C)C(C)(C)C
6	alkane	CCCCC(CCC)CCCC
7	alkane	CCCC(CC)CC(C)(C)C
8	alkane	CCCCCCCC(C)(C)C
9	alkane	CCC(C)CC(CC)CC(C)C
10	alkane	CCCC(C)CC(C)C
11	alkane	CCC(C)C(C)(C)CC
12	benzene derivative	Clc1ccccc1
13	alkane	CCCCCC(C)C
14	alcohol	CCC(C)C(C)O
15	alkane	CCCC(CCC)CCC
16	alkane	CCCCCC
17	alkane	CCCCC(C)(C)CC(C)(C)C
18	cycloalkane	C1CCCCC1
19	alkane	CC(C)(C)CCCCC(C)(C)C
20	alkane	CCCCCC(CC)CCCC
21	aldehyde	CCC=O
22	alkane	CCC(C)(C)CC

Continued on next page

Table SI 1 – Continued from previous page

NO.	SMILES	Category
23	alkane	CCCCC
24	alkane	CCC(CC)C(C)(C)C
25	alkane	CCCCCCC
26	alkane	CCC(CC)CC
27	alkane	CC(C)CC(C)C(C)(C)C
28	alkane	CC[C@@H](C)C(C)C
29	alkane	CCC(C)C
30	alkane	CCCCCC(C)CC
31	alkane	CC(C)C(C)(C)C(C)(C)C
32	alcohol	CC(C)C(C)O
33	alkane	CCCCCCCCC(C)(C)C
34	benzene derivative	NCc1ccccc1
35	alcohol	CC(C)(C)O
36	alcohol	CCCCCO
37	alkane	CC(C)CC(C)(C)C
38	alcohol	CCC(O)CC
39	alcohol	CCCO
40	alkane	CCCCCCCCC
41	alcohol	CC[C@@H](C)CO
42	alcohol	CCC[C@@H](C)O
43	alcohol	CCCCO
44	alkane	CCCC(C)(C)C
45	alkane	CCCCC(C)CC
46	alkane	CCCCC[C@@H](C)CC(C)C

Continued on next page

Table SI 1 – Continued from previous page

NO.	SMILES	Category
47	alkane	CC(C)C(C(C)C)C(C)C
48	alkane	CCC(C)CCC(C)CC
49	alcohol	CC(C)CCO
50	ketone	CC(=O)C(C)C
51	benzene derivative	O=[N+]([O-])c1ccccc1
52	aldehyde	CC(C)CC(C)C=O
53	alkane	CCC(C)C(C)(C)C(C)(C)C
54	alkane	CCCCC(C)C
55	alkane	CCCCCCCCC(C)C
56	alkane	CC(C)CCCCCC(C)C
57	alkane	CCCCCCCCCC(C)C
58	alkane	CCCCC(C)C(C)CC
59	alkane	CCC(C)CCCC(C)C
60	alkane	CCC(CC)C(C)C(CC)CC
61	alkane	CCC(C)C(C)(C)C
62	alcohol	CC(C)C(C)C(C)O
63	alkane	CCCC(C)CC
64	alkane	CCC(C)(C)C(C)C
65	alcohol	CC[C@H](C)CO
66	ketone	CCCCCCCC(=O)CC
67	alkane	CCC(CC)(CC)CC(C)C
68	alkane	CCCCCCC(C)(C)C
69	alkane	CCCCC(C)(C)C
70	alkane	CCCC(C)(C)CC

Continued on next page

Table SI 1 – Continued from previous page

NO.	SMILES	Category
71	alkane	CCCCCCC(C)C(C)CC
72	alkane	CCC(CC)C(C)C
73	alkane	CCCCCCCCCCC
74	alkane	CCCCCCC(C)CC
75	ketone	CCC(C)=O
76	alkane	CC(C)C(C)C(C)(C)C
77	alcohol	CC(C)CO
78	benzene derivative	CCc1ccccc1
79	alkane	CCC(CC)(CC)C(C)(C)C
80	alkane	CCCC(CC)CC
81	alkane	CCCC(CC)(CC)CC
82	benzene derivative	Cc1ccccc1
83	alkane	CC[C@H](C)C(C)C
84	ketone	CCCCCC(=O)CCCC
85	alkane	CCCCC(C)C(C)C
86	alkane	CCCC(C)CCC
87	alkane	CCC(C)(C)CC(C)CC(C)C
88	benzene derivative	c1ccccc1
89	aldehyde	CCCC=O
90	alkane	CCCCCCCCC(C)CC
91	alkane	CC(C)C(C)C(C)(C)C(C)(C)C
92	alkane	CCCCCCCC
93	alkane	CCCCCC(C)(C)C
94	alkane	CC(C(C)(C)C)C(C)(C)C

Continued on next page

Table SI 1 – Continued from previous page

NO.	SMILES	Category
95	alkane	CCC(CC)(CC)CC
96	alkane	CCC(C)(C)C
97	alkane	CC(C)(C)C(C)(C)C
98	alcohol	CC(C)(C)CO
99	alkane	CCC(C)(C)C(CC)(CC)CC
100	alcohol	CCC(C)(C)O
101	alkane	CC(C)CC(C)C
102	aldehyde	CC=O
103	alkane	CCC(C)(C)C(C)(C)CC
104	alkane	CC(C)C(C)C
105	alkane	CCC(C)C(C)(CC)C(C)(C)C
106	alkane	CC(CC(C)(C)C)C(C)(C)C
107	alkane	CCC(C)C(C)C(C)C(C)CC
108	aldehyde	CC(C)CC=O
109	alkane	CCCC(C)(CC)CC
110	alkane	CCCCCCCCCCC
111	alkane	CCCCC(C)C(C)CCCC
112	aldehyde	CC(C)C(C)C(C)(C)C=O
113	ketone	CCCC(=O)CC
114	alkane	CCCCCCC(C)CCCC
115	aldehyde	CC(C)C(C)C(C)C=O
116	alkane	CCC(C)CC
117	alkane	CC(C)CCCC(C)C
118	alcohol	CC(C)O

Continued on next page

Table SI 1 – Continued from previous page

NO.	SMILES	Category
119	alkane	CCC(C)(CC)C(C)(C)C
120	aldehyde	CC(C)CCCCC=O
121	alkane	CCC(CC)C(CC)CC
122	alkane	CC(C)(C)CC(C)(C)C
123	alkane	CCC(C)CC(C)(C)CC
124	alkane	CC(C)C(C)(C)C
125	alkane	CC(C)CCC(C)(C)C
126	alkane	CCCC(C)C
127	alkane	CC(C)CCC(C)C
128	alkane	CC(C)CC(C)(C)CC(C)C
129	alcohol	CC[C@@H](C)O
130	alkane	CC[C@@H](C)[C@@H](C)CC
131	aldehyde	CC(C)C(C)C=O
132	alkane	CCC(C)(C)C(C)(C)C
133	alkane	CC(C)C(C)C(C)C
134	alkane	CCCCCCC(C)C
135	alkane	CC(C)(C)C
136	alkane	CCCCC(CC)CCCC
137	cycloalkane	C1CCCC1
138	alkane	CCC(C)(C(C)C)C(C)(C)C
139	alcohol	CC(C)C(C)(C)O
140	alkane	CCCCCCC(C)CC
141	alcohol	CC[C@H](C)O
142	alkane	CCCCCCCCCC

Continued on next page

Table SI 1 – Continued from previous page

**NO.**	**SMILES**	**Category**
143	alkane	CC[C@@H](C)[C@@H](C)C(C)C
144	ketone	CCCCC(C)=O
145	ketone	CCCCC(=O)CCC

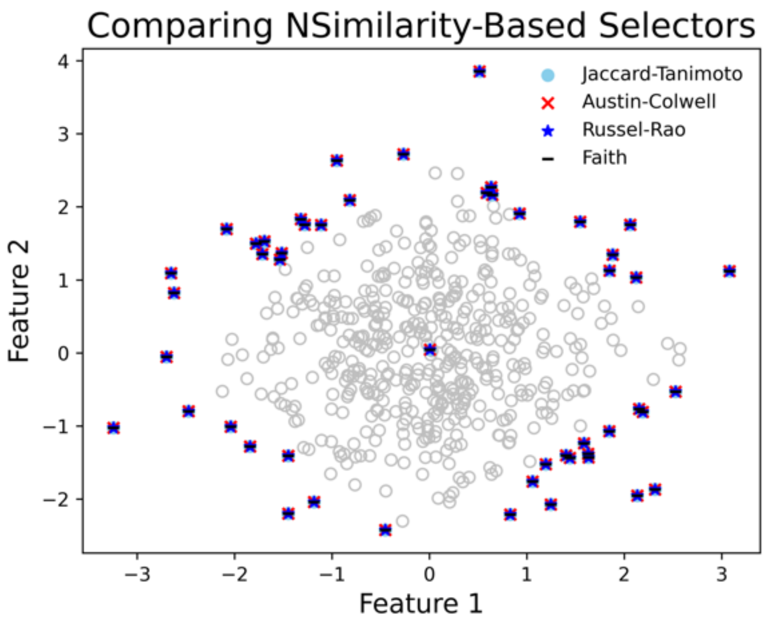

Fig. SI 2 Comparing NSimilarity based selection methods.

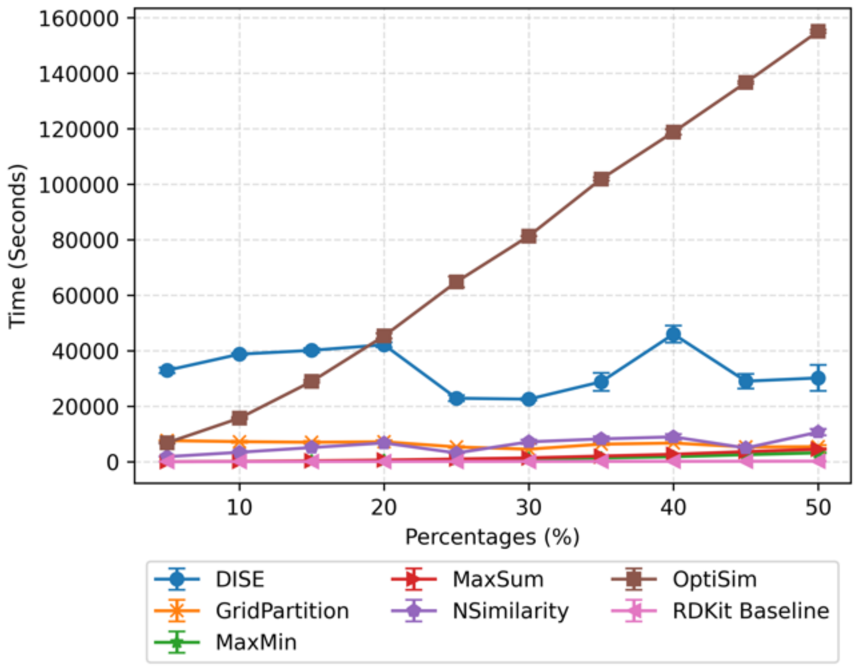

Fig. SI 3 Running time comparisons of different subset selection algorithms. The 1024-bit ECFP4 fingerprints were computed with `RDKit` for the 14k logP dataset from EPA public data[1]. The `MaxMinPicker` method in `RDKit` was as the base line. It is noticed OptiSim are slower because the setup of searching the optimal radius in `Selector` in order to get the required number of selected data points.

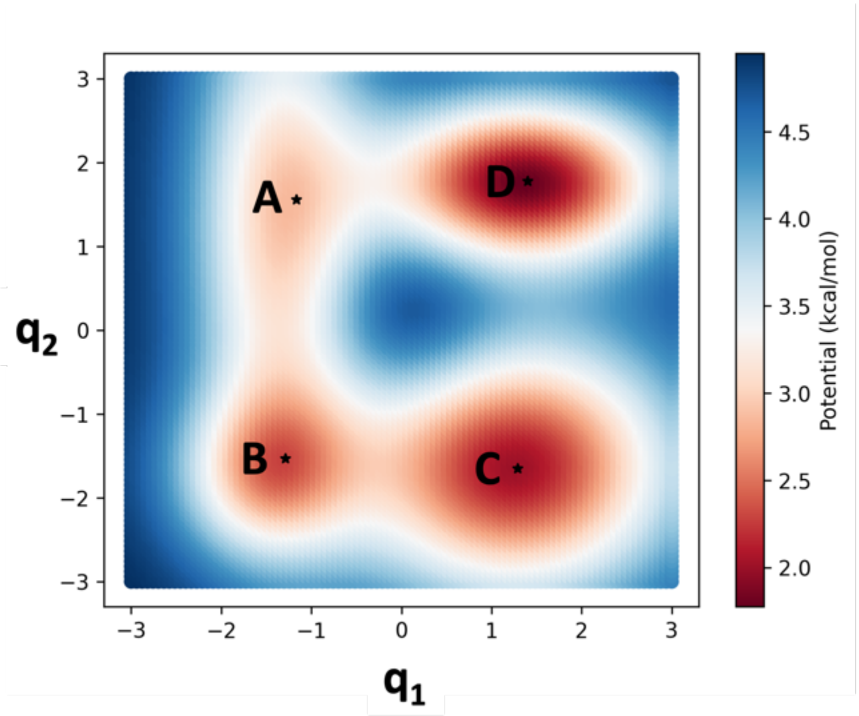

Fig. SI 4 The four-well potential with four minima: $V_A(-1.17, 1.56) = 2.84$, $V_B(-1.29, -1.53) = 2.28$, $V_C(1.29, -1.65) = 2.01$, and $V_D(1.4, 1.78) = 1.77$ kcal/mol.[2]

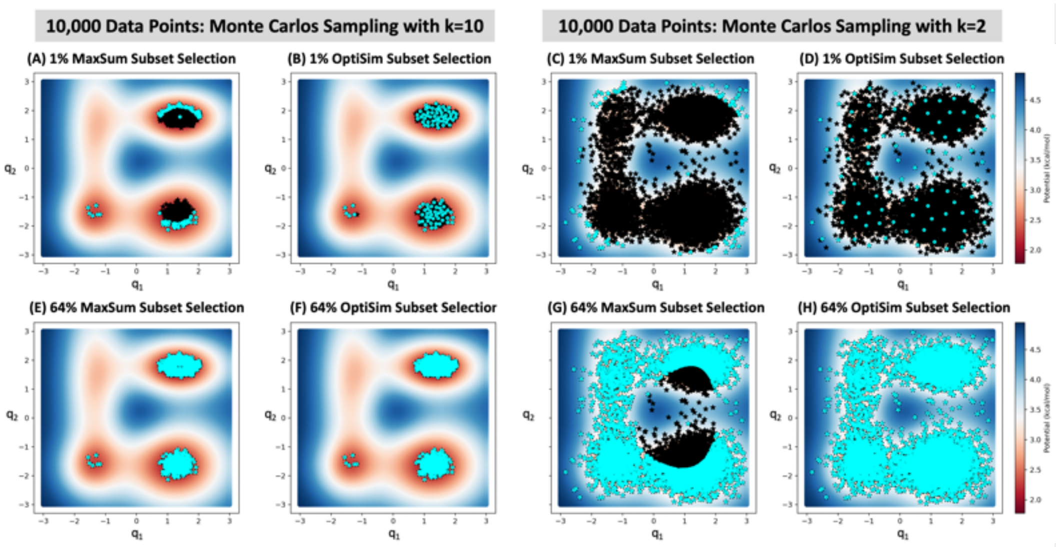

Fig. SI 5 Selecting subsets (colored with cyan circles) of four-well potential with MaxSum (1st and 3rd columns) and OptiSim (2nd and 4th columns) methods from a dataset of 10,000 points (colored with black stars) generated with Monte Carlo sampling using values of k=10 (two left columns) and k=2 (two right columns).

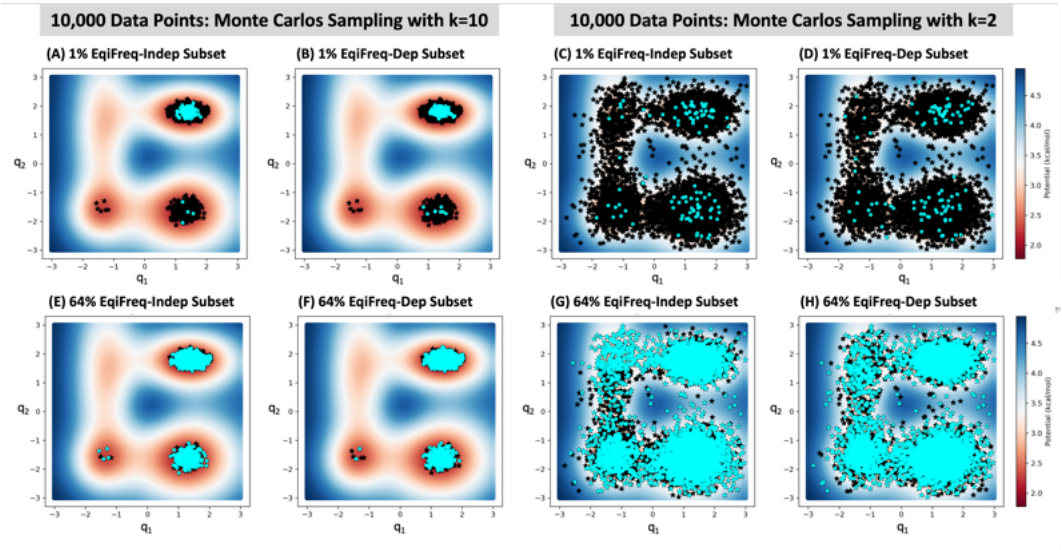

Fig. SI 6 Selecting subsets (colored with cyan circles) of four-well potential with Grid Partition Equifrequent Dependent (1st and 3rd columns) and Independent (2nd and 4th columns) methods from a dataset of 10,000 points (colored with black stars) generated with Monte Carlo sampling using values of k=10 (two left columns) and k=2 (two right columns). For the Grid Partition, 5 bins are used along each access.

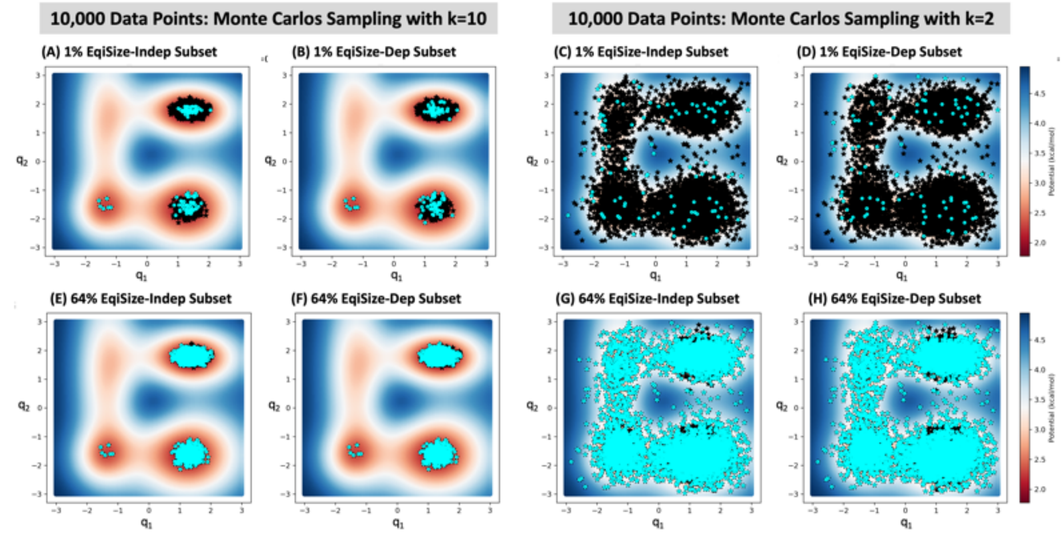

Fig. SI 7 Selecting subsets (colored with cyan circles) of four-well potential with Grid Partition Equisize Dependent (1st and 3rd columns) and Independent (2nd and 4th columns) methods from a dataset of 10,000 points (colored with black stars) generated with Monte Carlo sampling using values of k=10 (two left columns) and k=2 (two right columns). For the Grid Partition, 5 bins are used along each access.

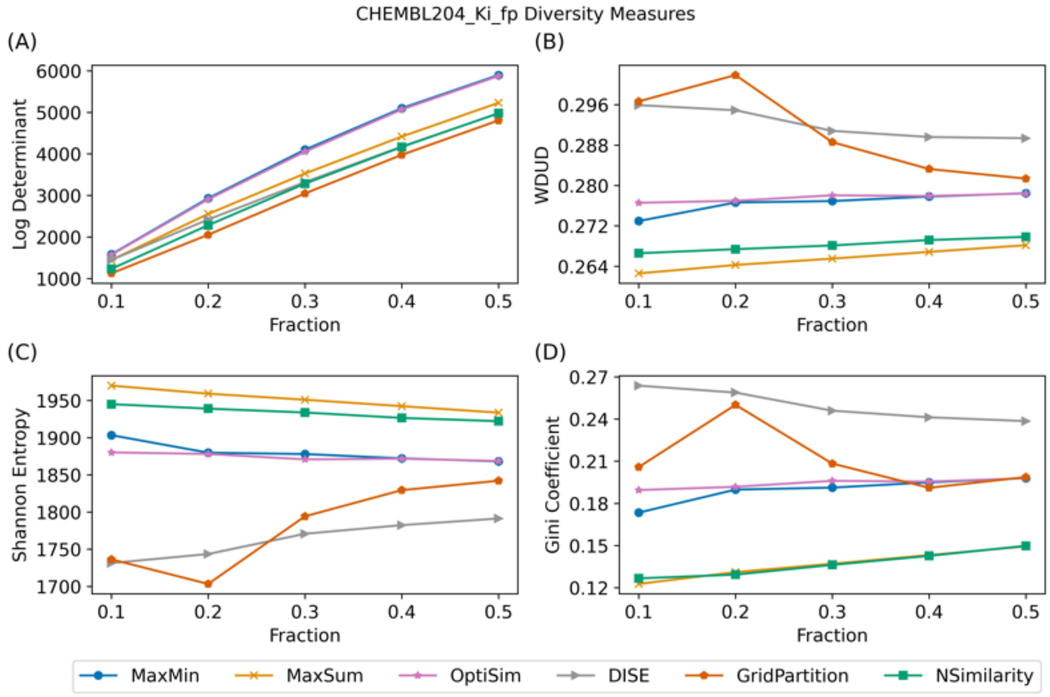

Fig. SI 8 Diversity selection measurements of ChEMBL204 Ki subset ($n_{total} = 2754$) selection with different selection methods. (A) Log-determinant of the selected subset. (B) Wasserstein distance to uniform distribution (WDUD) of the selected subset. (C) Shannon entropy of the selected subset. (D) Gini coefficient of the selected subset. Based on the WDUD, MaxSum and NSimilarity perform the best across the different fractions. Grid partitioning has the lowest diversity when less than 30% of the data is selected, but for larger fractions of the data DISE performs worst. The Gini coefficient, MaxSum and NSimilarity consistently yield the most diverse sets and DISE consistently gives the least diverse set.

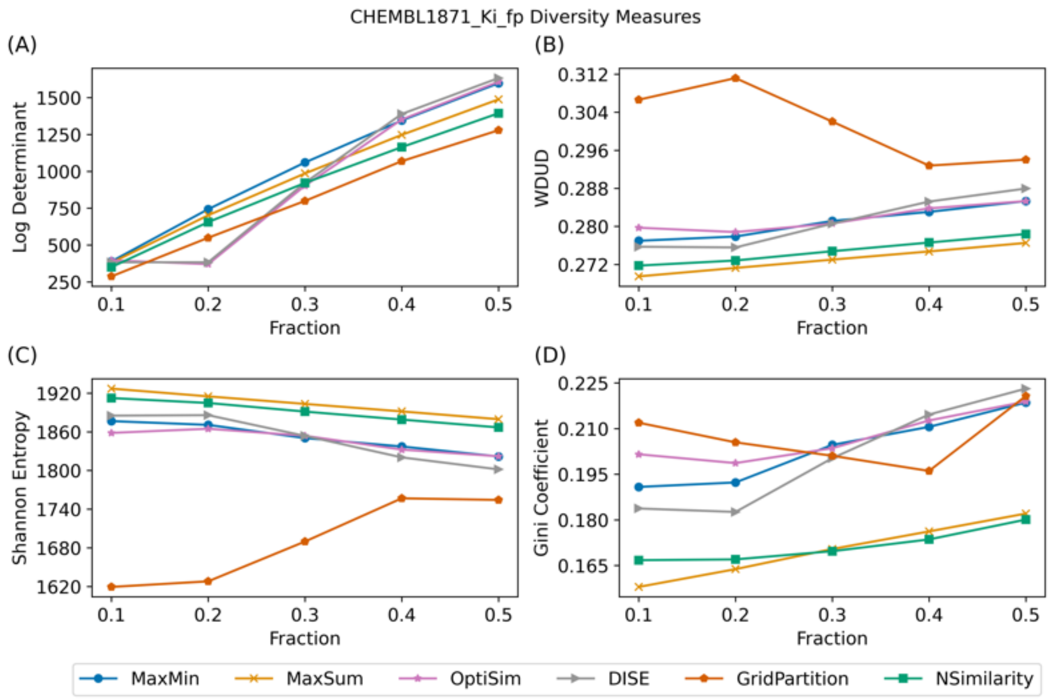

Fig. SI 9 Diversity selection measurements of ChEMBL1871 Ki subset ($n_{total} = 659$) selection with different selection methods. (A) Log-determinant of the selected subset. (B) Wasserstein distance to uniform distribution (WDUD) of the selected subset. (C) Shannon entropy of the selected subset. (D) Gini coefficient of the selected subset.

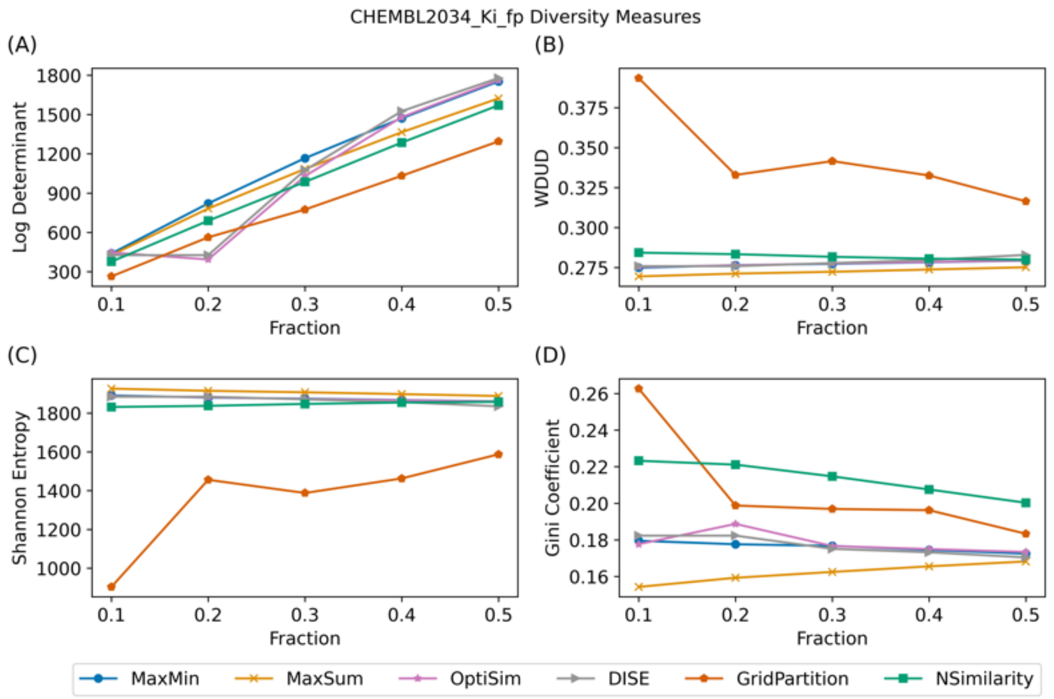

Fig. SI 10 Diversity selection measurements of ChEMBL2034 Ki subset ($n_{total} = 750$) selection with different selection methods. (A) Log-determinant of the selected subset. (B) Wasserstein distance to uniform distribution (WDUD) of the selected subset. (C) Shannon entropy of the selected subset. (D) Gini coefficient of the selected subset.

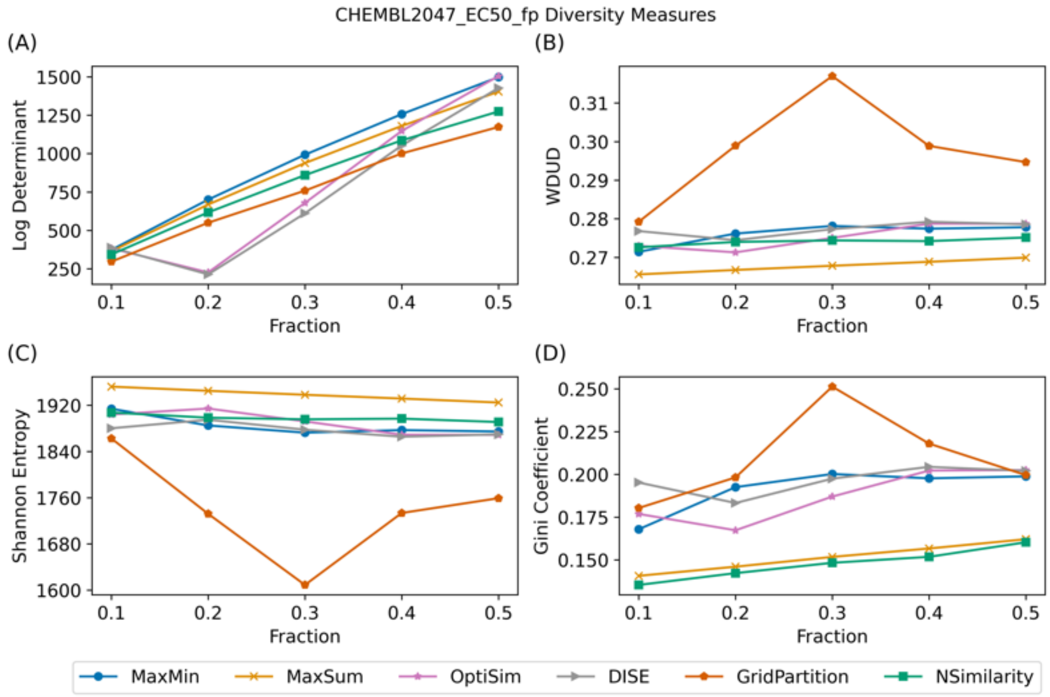

Fig. SI 11 Diversity selection measurements of ChEMBL2047 EC50 subset ($n_{total} = 631$) selection with different selection methods. (A) Log-determinant of the selected subset. (B) Wasserstein distance to uniform distribution (WDUD) of the selected subset. (C) Shannon entropy of the selected subset. (D) Gini coefficient of the selected subset.

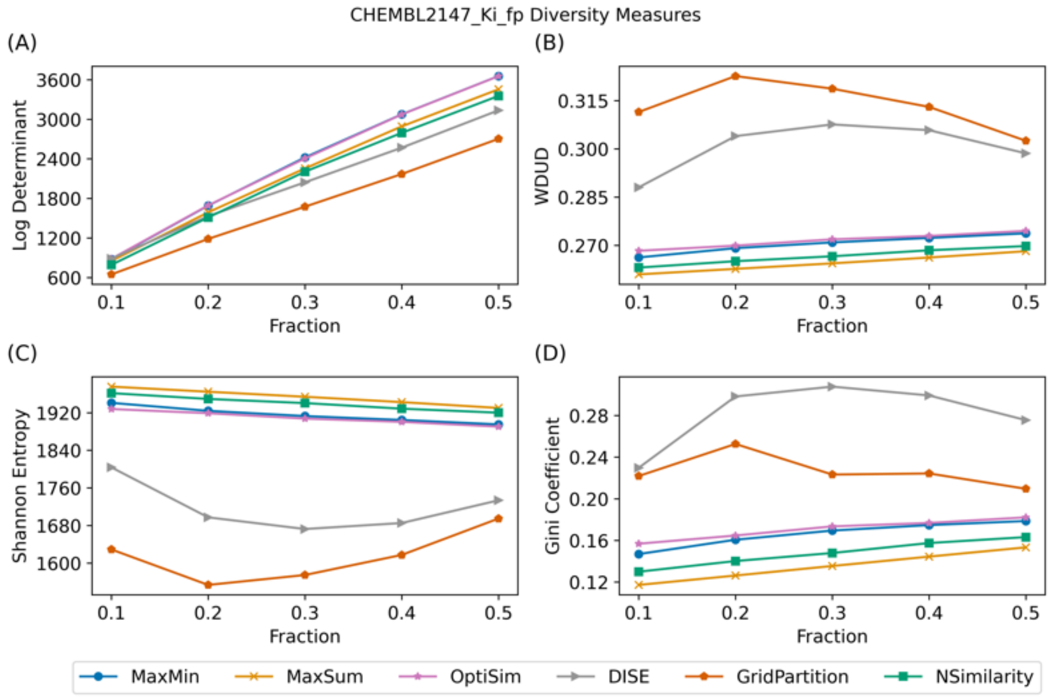

Fig. SI 12 Diversity selection measurements of ChEMBL2147 Ki subset ($n_{total} = 1456$) selection with different selection methods. (A) Log-determinant of the selected subset. (B) Wasserstein distance to uniform distribution (WDUD) of the selected subset. (C) Shannon entropy of the selected subset. (D) Gini coefficient of the selected subset.

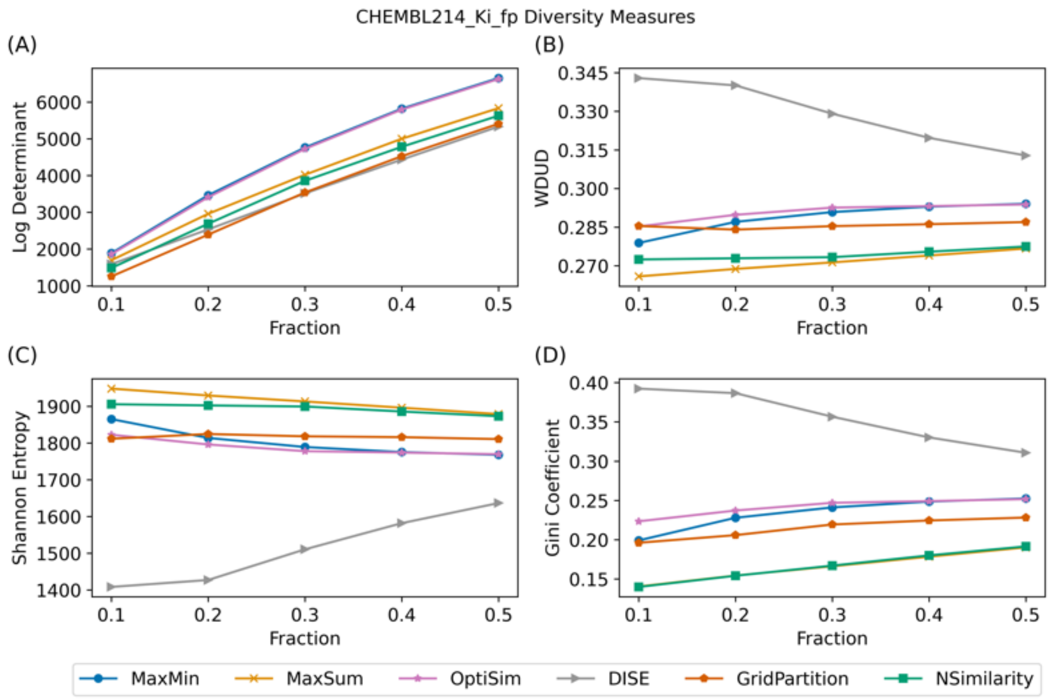

Fig. SI 13 Diversity selection measurements of ChEMBL214 Ki subset ($n_{total} = 3317$) selection with different selection methods. (A) Log-determinant of the selected subset. (B) Wasserstein distance to uniform distribution (WDUD) of the selected subset. (C) Shannon entropy of the selected subset. (D) Gini coefficient of the selected subset.

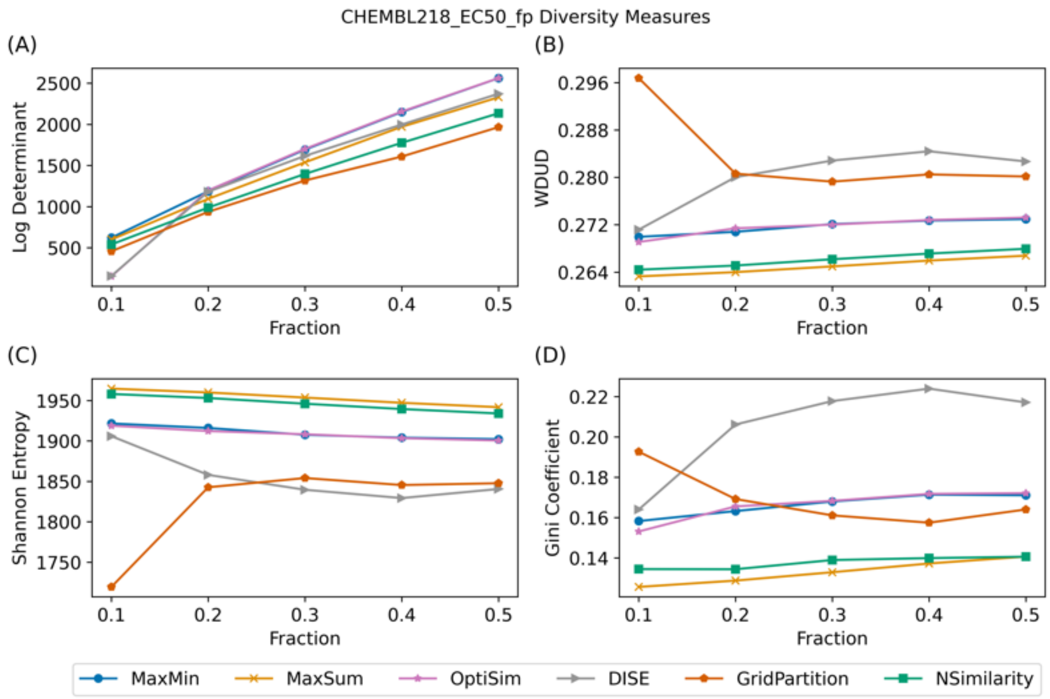

Fig. SI 14 Diversity selection measurements of ChEMBL218 EC50 subset ($n_{total} = 1031$) selection with different selection methods. (A) Log-determinant of the selected subset. (B) Wasserstein distance to uniform distribution (WDUD) of the selected subset. (C) Shannon entropy of the selected subset. (D) Gini coefficient of the selected subset.

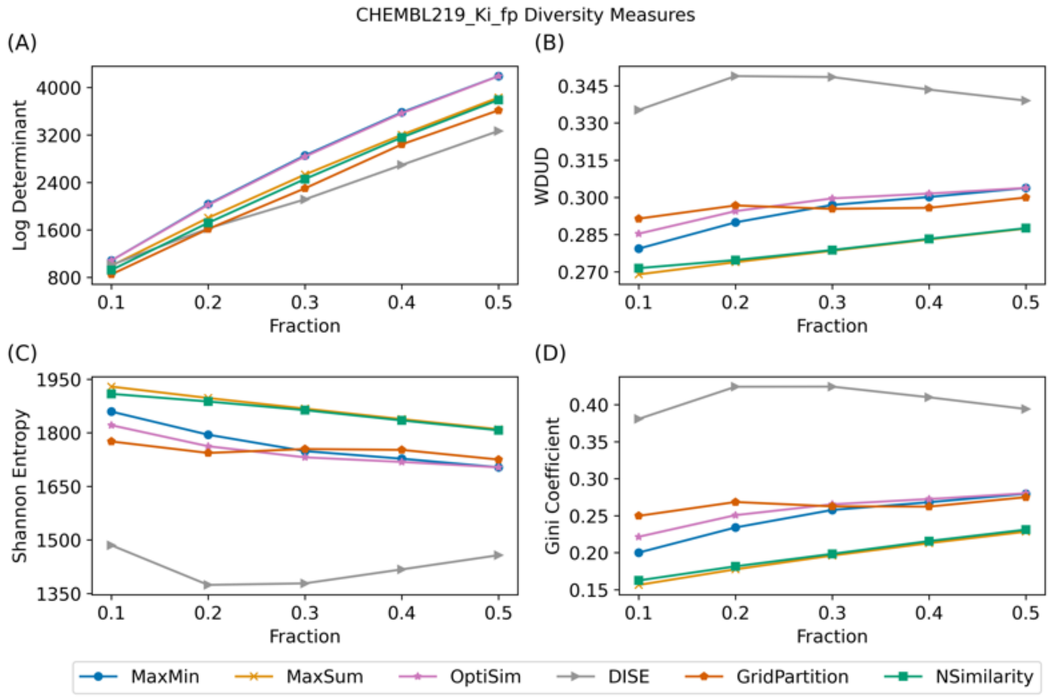

Fig. SI 15 Diversity selection measurements of ChEMBL219 Ki subset ($n_{total} = 1859$) selection with different selection methods. (A) Log-determinant of the selected subset. (B) Wasserstein distance to uniform distribution (WDUD) of the selected subset. (C) Shannon entropy of the selected subset. (D) Gini coefficient of the selected subset.

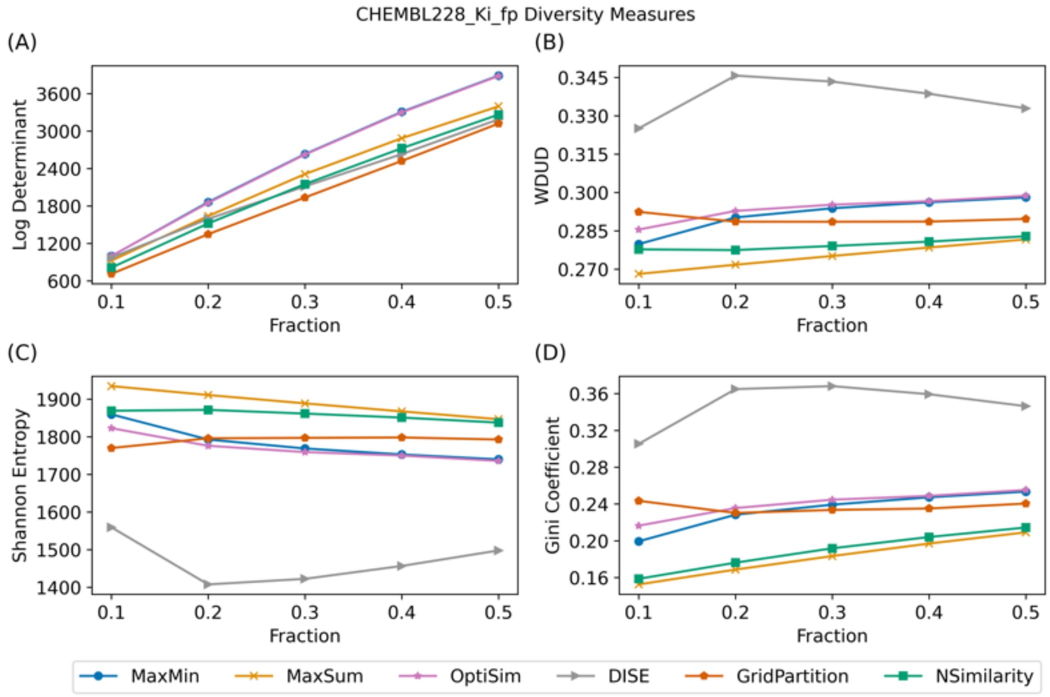

Fig. SI 16 Diversity selection measurements of ChEMBL228 Ki subset ($n_{total} = 1704$) selection with different selection methods.

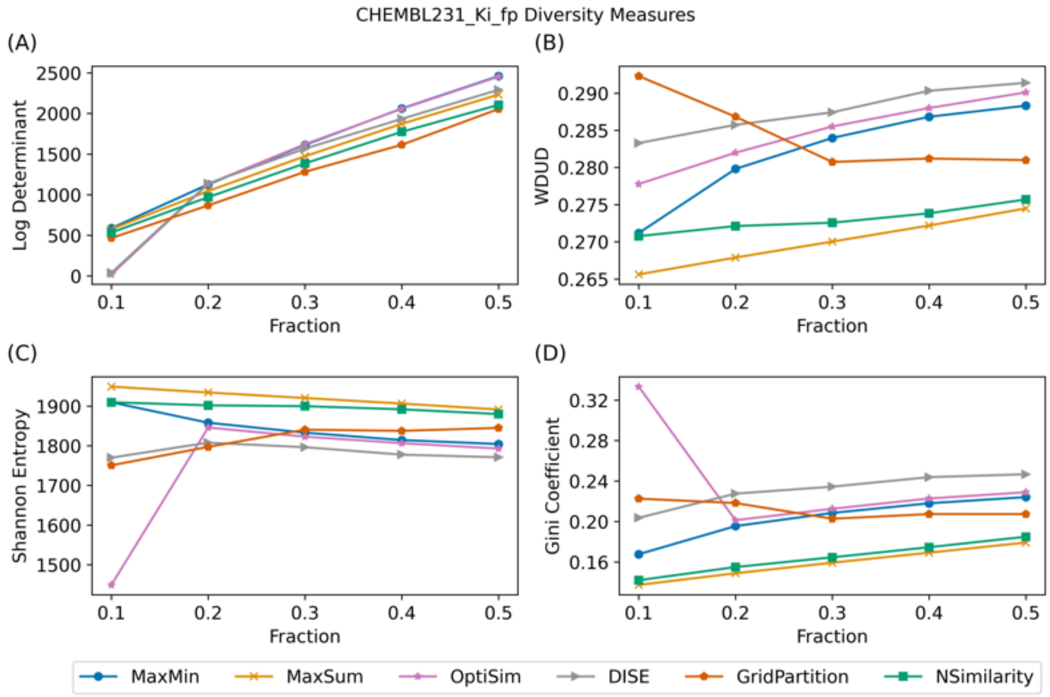

Fig. SI 17 Diversity selection measurements of ChEMBL231 Ki subset ($n_{total} = 973$) selection with different selection methods. (A) Log-determinant of the selected subset. (B) Wasserstein distance to uniform distribution (WDUD) of the selected subset. (C) Shannon entropy of the selected subset. (D) Gini coefficient of the selected subset.

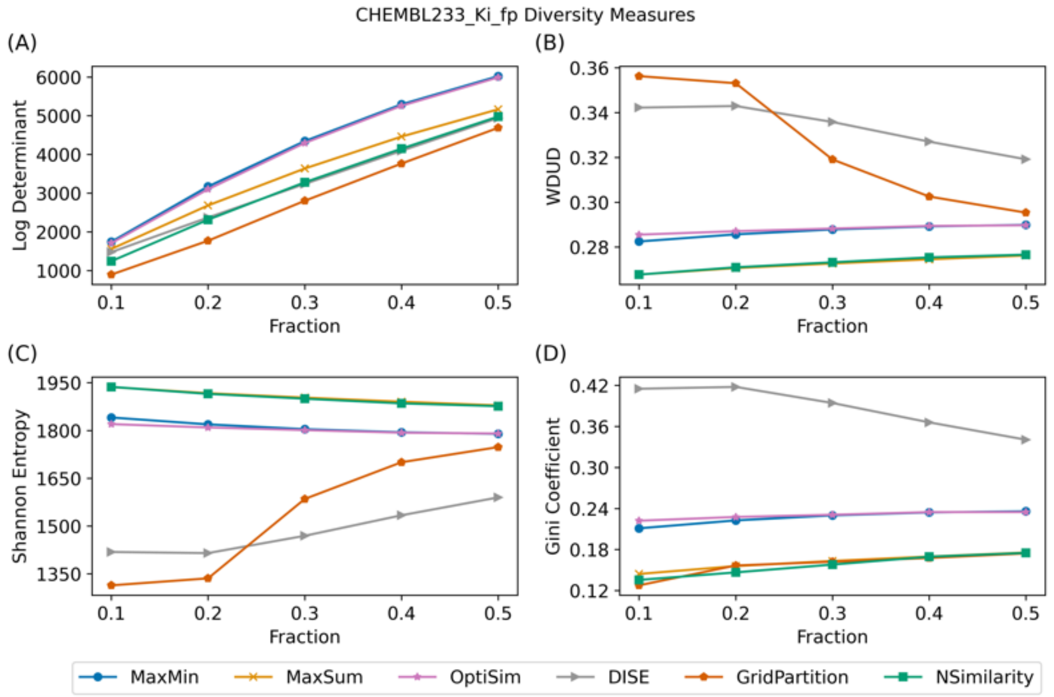

Fig. SI 18 Diversity selection measurements of ChEMBL233 Ki subset ($n_{total} = 3142$) selection with different selection methods. (A) Log-determinant of the selected subset. (B) Wasserstein distance to uniform distribution (WDUD) of the selected subset. (C) Shannon entropy of the selected subset. (D) Gini coefficient of the selected subset.

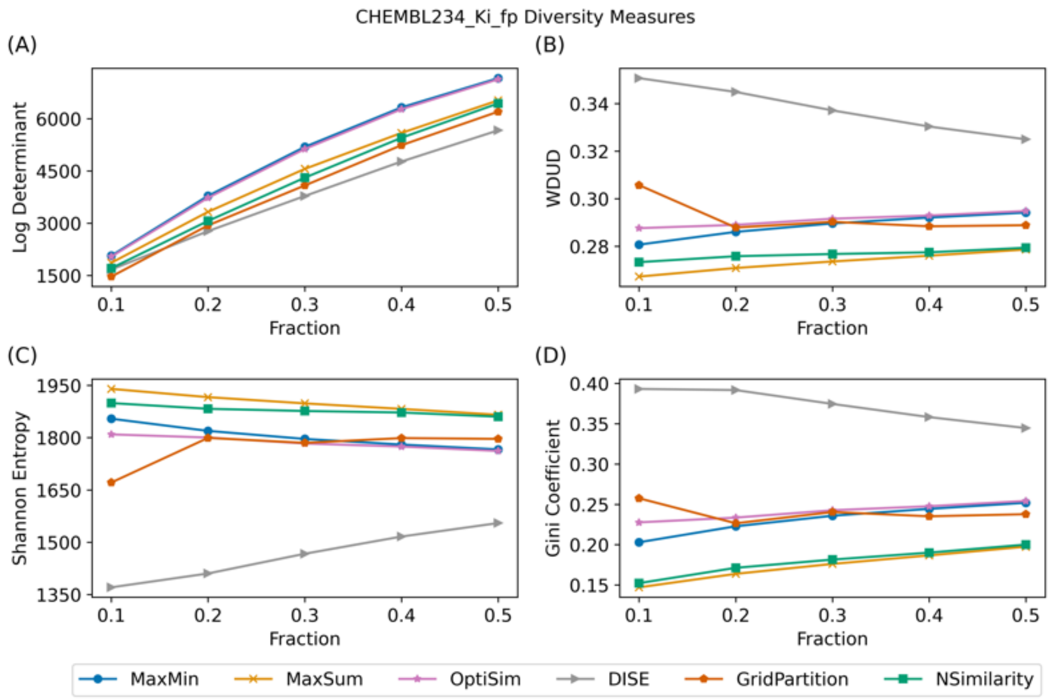

Fig. SI 19 Diversity selection measurements of ChEMBL234 Ki subset ($n_{total} = 3657$) selection with different selection methods. (A) Log-determinant of the selected subset. (B) Wasserstein distance to uniform distribution (WDUD) of the selected subset. (C) Shannon entropy of the selected subset. (D) Gini coefficient of the selected subset.

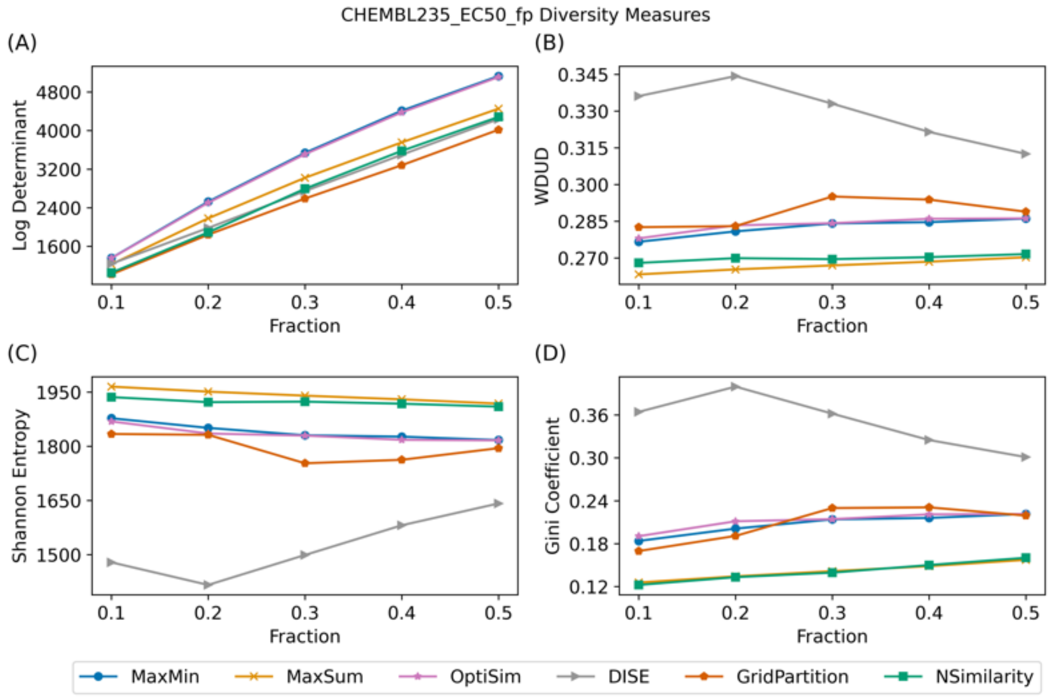

Fig. SI 20 Diversity selection measurements of ChEMBL235 EC50 subset ($n_{total} = 2349$) selection with different selection methods. (A) Log-determinant of the selected subset. (B) Wasserstein distance to uniform distribution (WDUD) of the selected subset. (C) Shannon entropy of the selected subset. (D) Gini coefficient of the selected subset.

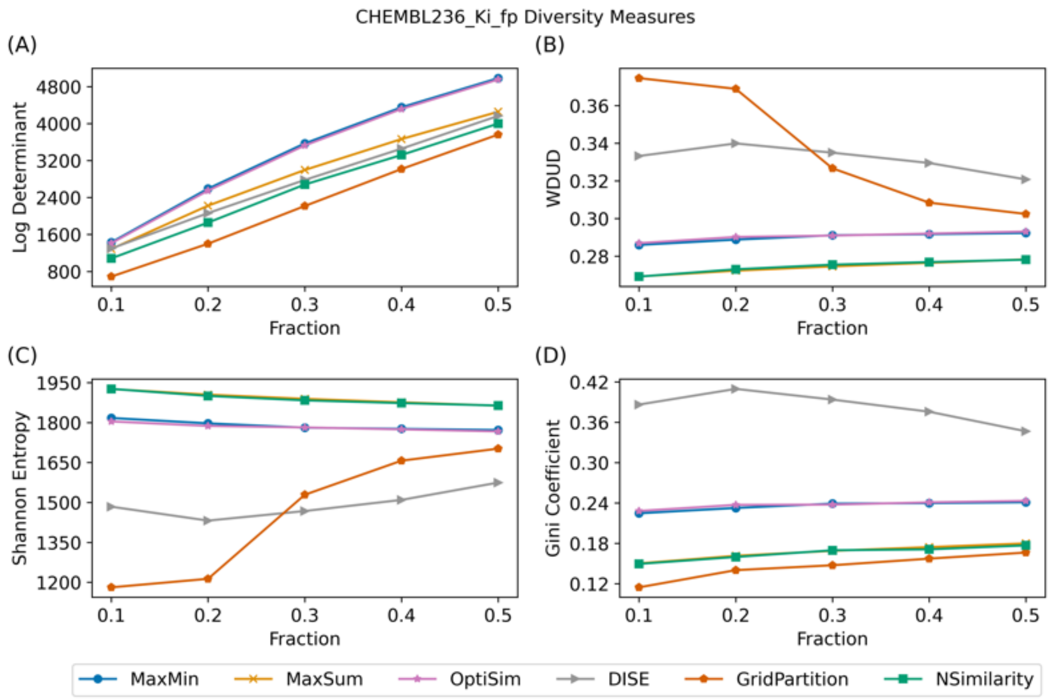

Fig. SI 21 Diversity selection measurements of ChEMBL236 Ki subset ($n_{total} = 2598$) selection with different selection methods. (A) Log-determinant of the selected subset. (B) Wasserstein distance to uniform distribution (WDUD) of the selected subset. (C) Shannon entropy of the selected subset. (D) Gini coefficient of the selected subset.

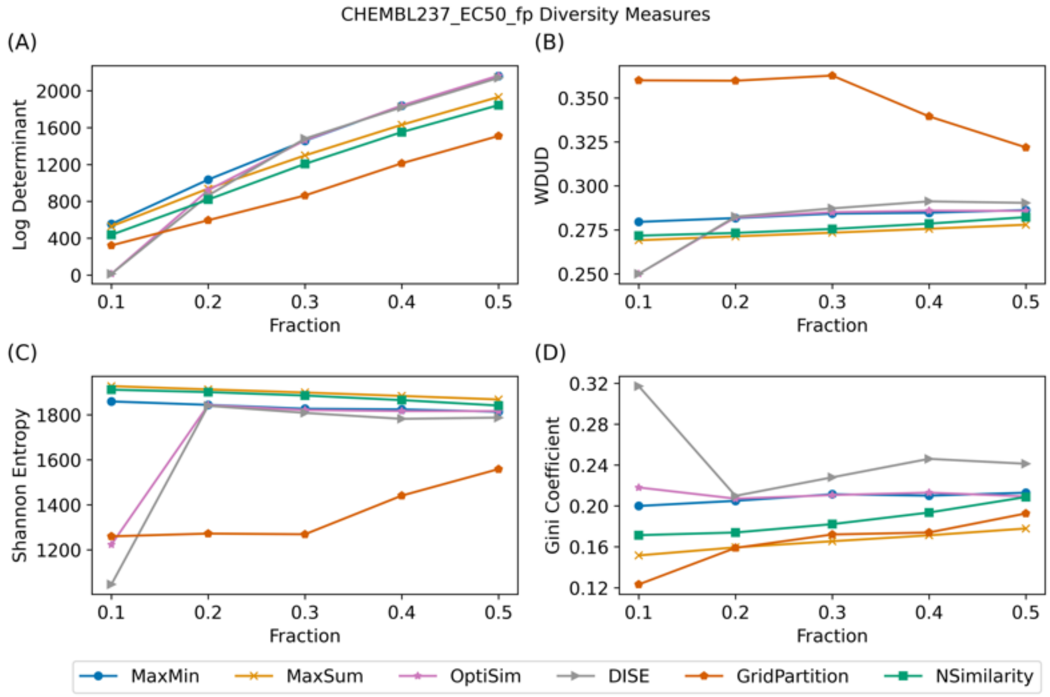

Fig. SI 22 Diversity selection measurements of ChEMBL237 EC50 subset ($n_{total} = 955$) selection with different selection methods.

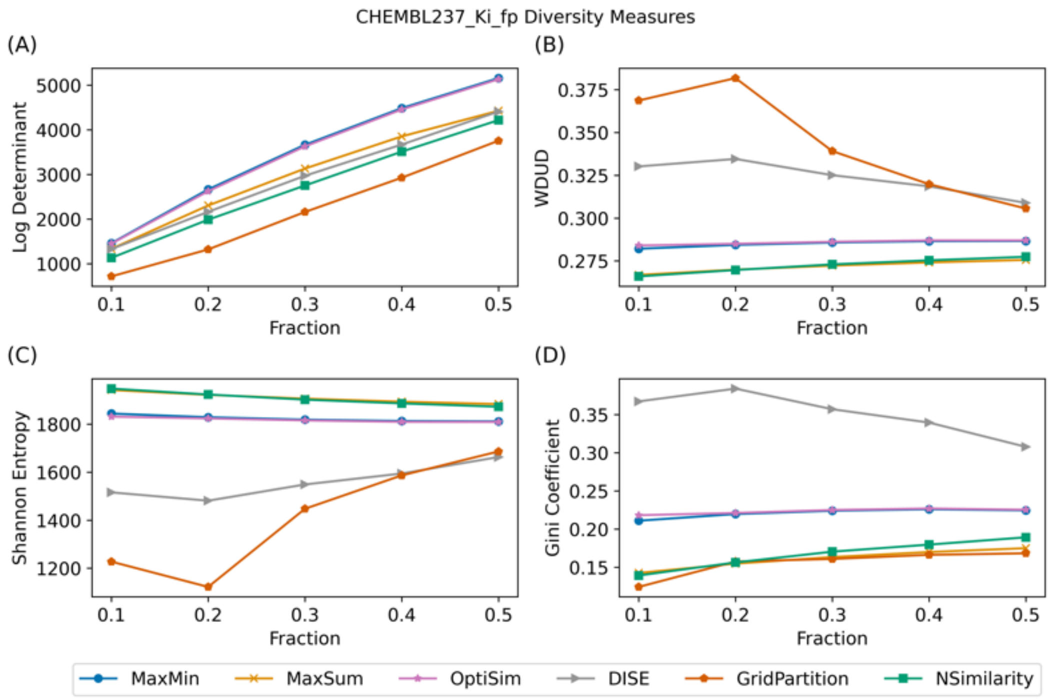

Fig. SI 23 Diversity selection measurements of ChEMBL237 Ki subset ($n_{total} = 2602$) selection with different selection methods. (A) Log-determinant of the selected subset. (B) Wasserstein distance to uniform distribution (WDUD) of the selected subset. (C) Shannon entropy of the selected subset. (D) Gini coefficient of the selected subset.

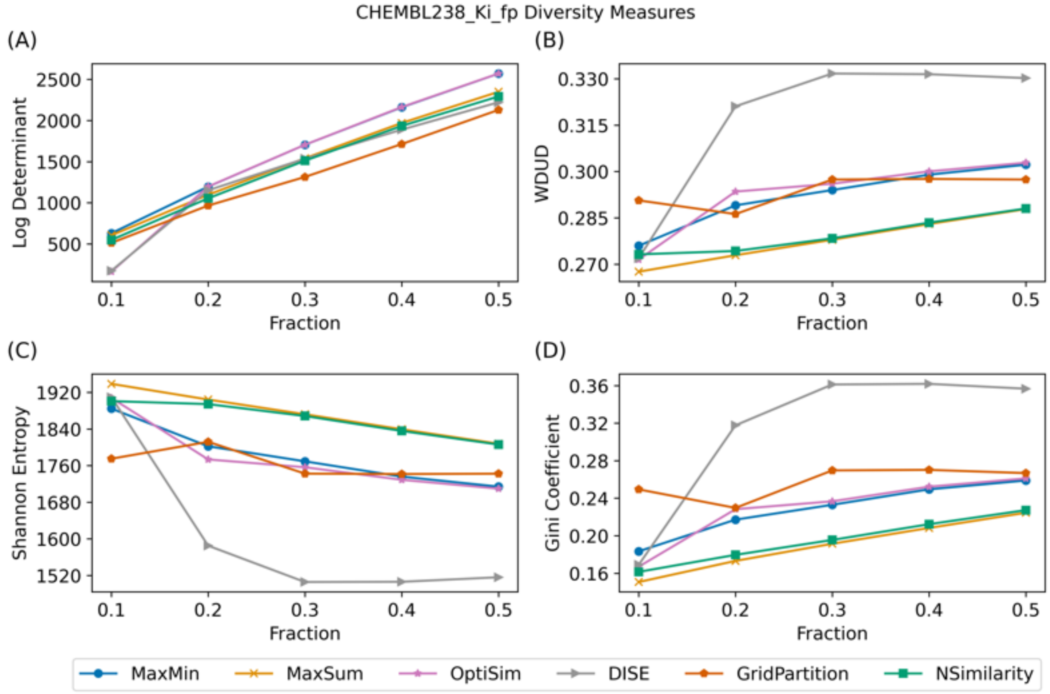

Fig. SI 24 Diversity selection measurements of ChEMBL238 Ki subset ($n_{total} = 1052$) selection with different selection methods. (A) Log-determinant of the selected subset. (B) Wasserstein distance to uniform distribution (WDUD) of the selected subset. (C) Shannon entropy of the selected subset. (D) Gini coefficient of the selected subset.

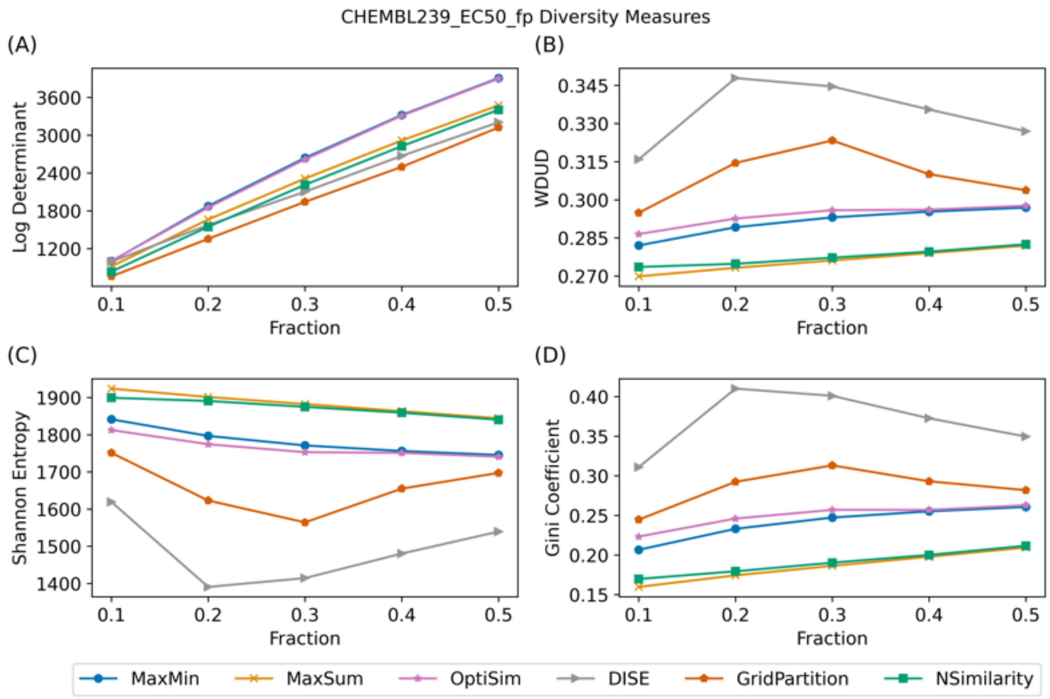

Fig. SI 25 Diversity selection measurements of ChEMBL239 EC50 subset ($n_{total} = 1721$) selection with different selection methods. (A) Log-determinant of the selected subset. (B) Wasserstein distance to uniform distribution (WDUD) of the selected subset. (C) Shannon entropy of the selected subset. (D) Gini coefficient of the selected subset.

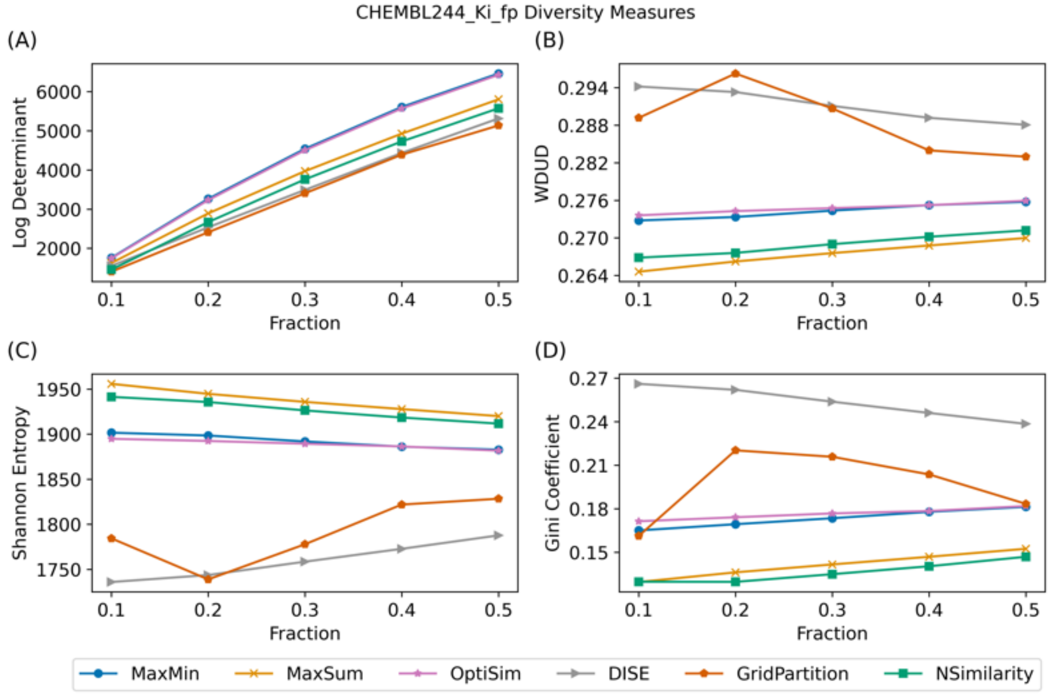

Fig. SI 26 Diversity selection measurements of ChEMBL244 Ki subset ($n_{total} = 3097$) selection with different selection methods. (A) Log-determinant of the selected subset. (B) Wasserstein distance to uniform distribution (WDUD) of the selected subset. (C) Shannon entropy of the selected subset. (D) Gini coefficient of the selected subset.

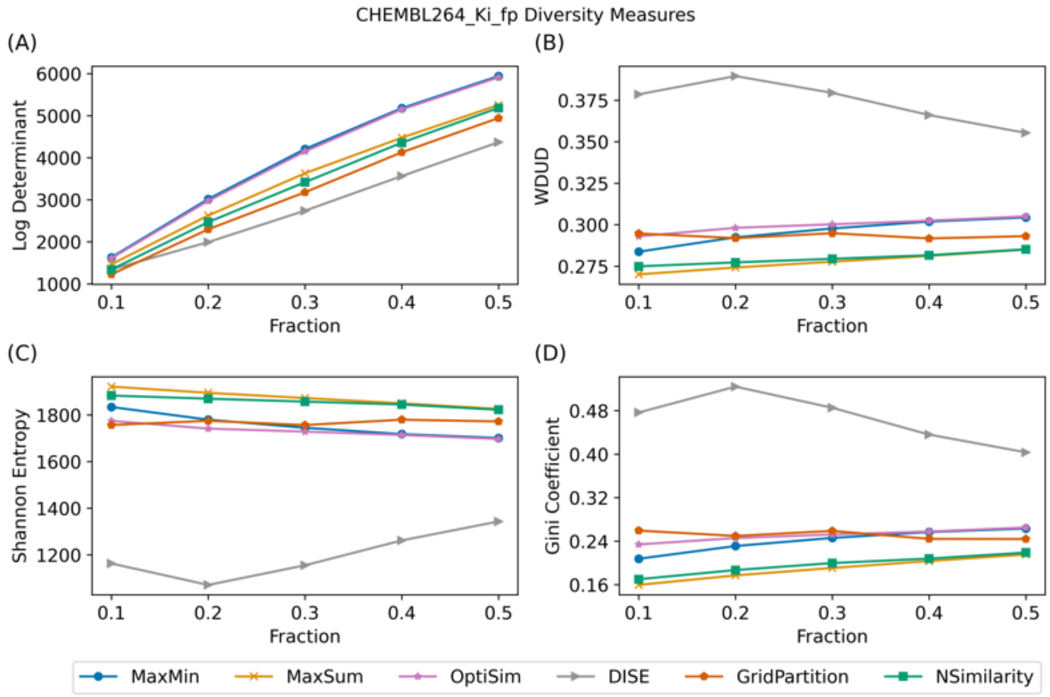

Fig. SI 27 Diversity selection measurements of ChEMBL264 Ki subset ($n_{total} = 2862$) selection with different selection methods. (A) Log-determinant of the selected subset. (B) Wasserstein distance to uniform distribution (WDUD) of the selected subset. (C) Shannon entropy of the selected subset. (D) Gini coefficient of the selected subset.

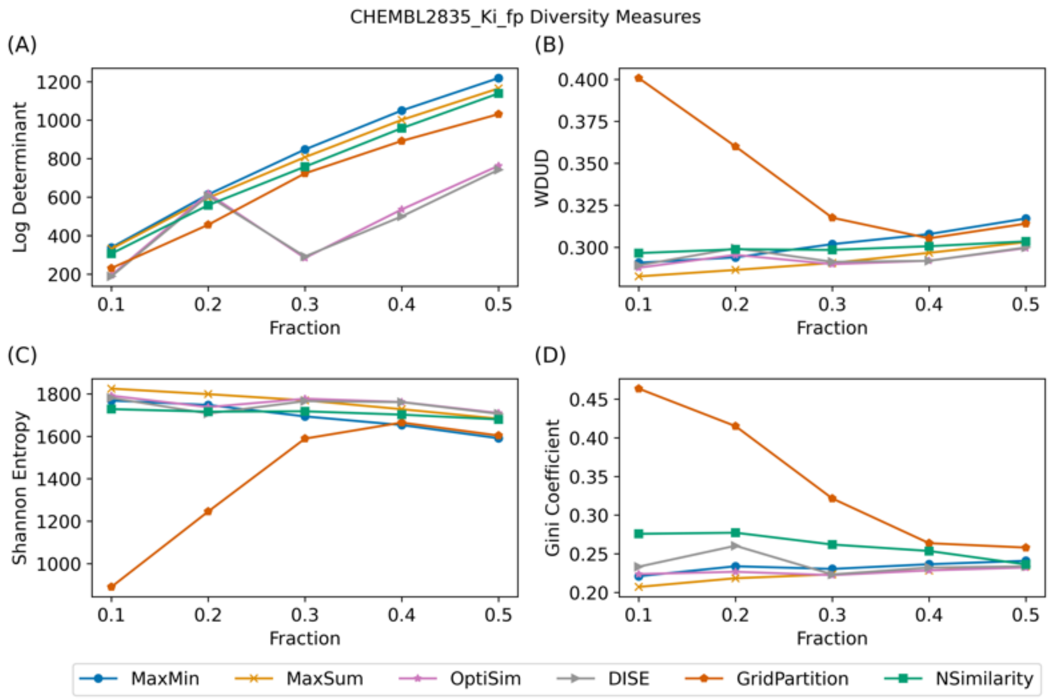

Fig. SI 28 Diversity selection measurements of ChEMBL2835 Ki subset ($n_{total} = 615$) selection with different selection methods. (A) Log-determinant of the selected subset. (B) Wasserstein distance to uniform distribution (WDUD) of the selected subset. (C) Shannon entropy of the selected subset. (D) Gini coefficient of the selected subset.

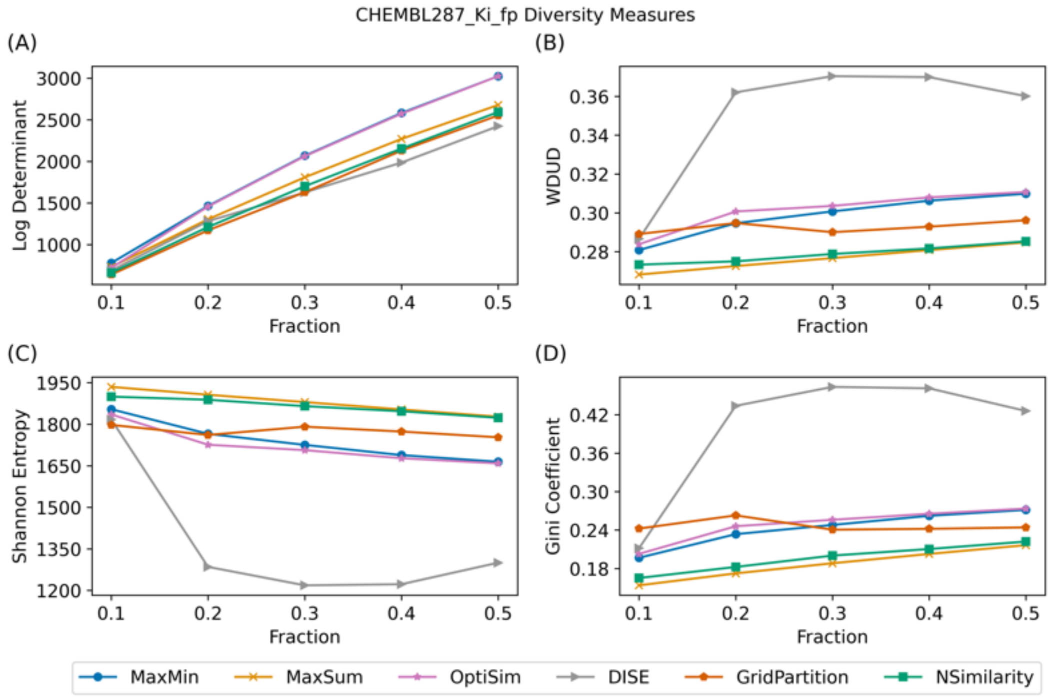

Fig. SI 29 Diversity selection measurements of ChEMBL287 Ki subset ($n_{total} = 1328$) selection with different selection methods. (A) Log-determinant of the selected subset. (B) Wasserstein distance to uniform distribution (WDUD) of the selected subset. (C) Shannon entropy of the selected subset. (D) Gini coefficient of the selected subset.

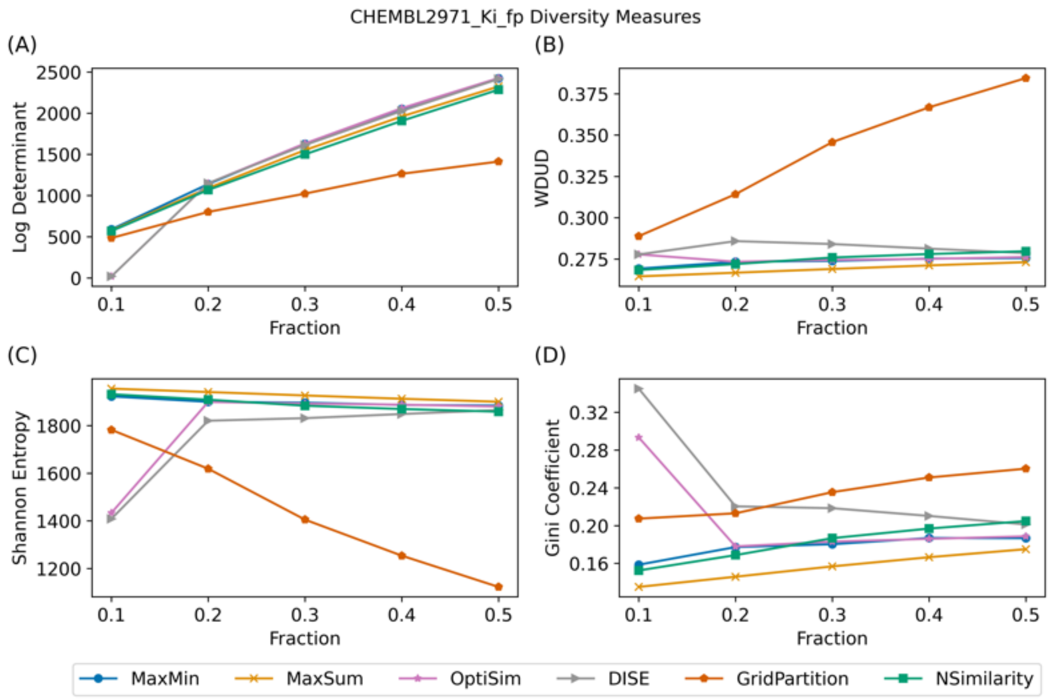

Fig. SI 30 Diversity selection measurements of ChEMBL2971 Ki subset ($n_{total} = 976$) selection with different selection methods. (A) Log-determinant of the selected subset. (B) Wasserstein distance to uniform distribution (WDUD) of the selected subset. (C) Shannon entropy of the selected subset. (D) Gini coefficient of the selected subset.

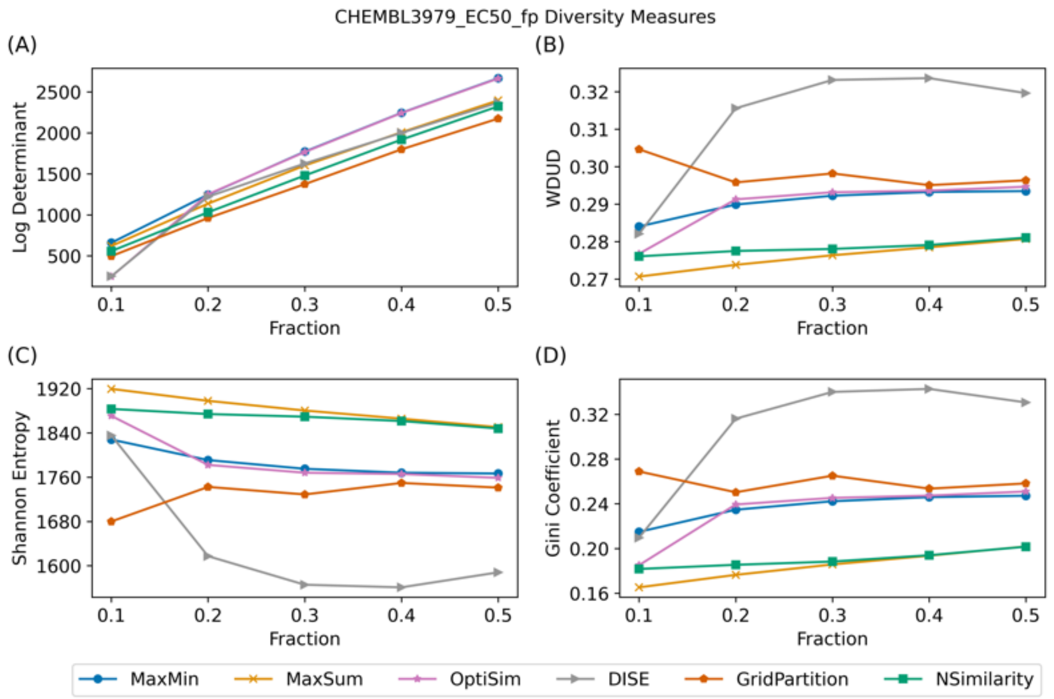

Fig. SI 31 Diversity selection measurements of ChEMBL3979 EC50 subset ($n_{total} = 1125$) selection with different selection methods. (A) Log-determinant of the selected subset. (B) Wasserstein distance to uniform distribution (WDUD) of the selected subset. (C) Shannon entropy of the selected subset. (D) Gini coefficient of the selected subset.

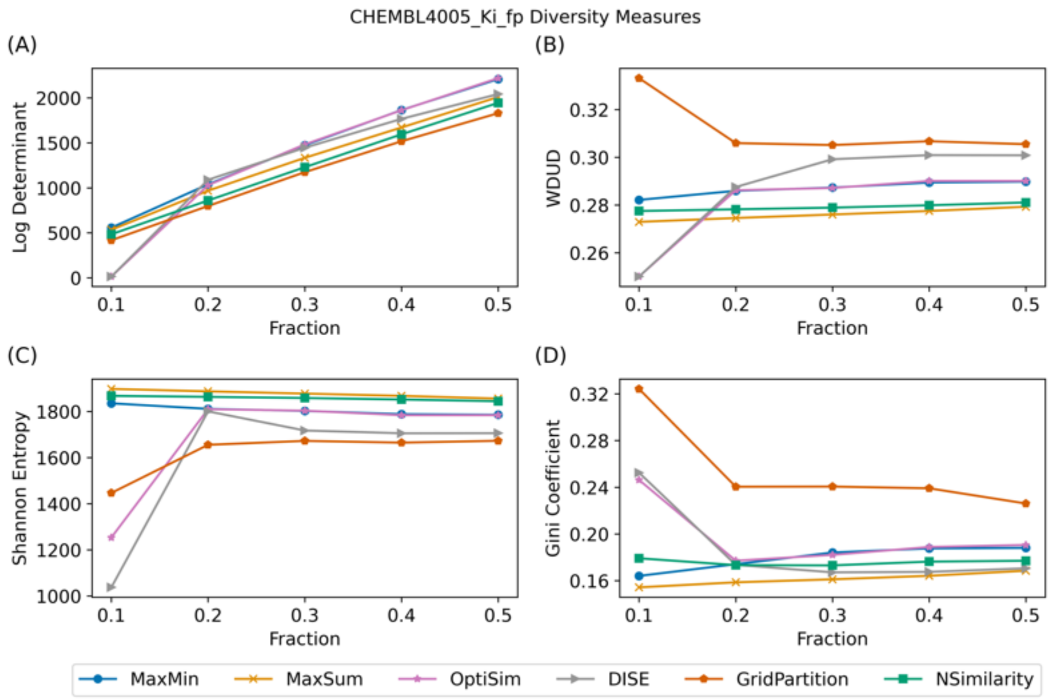

Fig. SI 32 Diversity selection measurements of ChEMBL4005 Ki subset ($n_{total} = 960$) selection with different selection methods. (A) Log-determinant of the selected subset. (B) Wasserstein distance to uniform distribution (WDUD) of the selected subset. (C) Shannon entropy of the selected subset. (D) Gini coefficient of the selected subset.

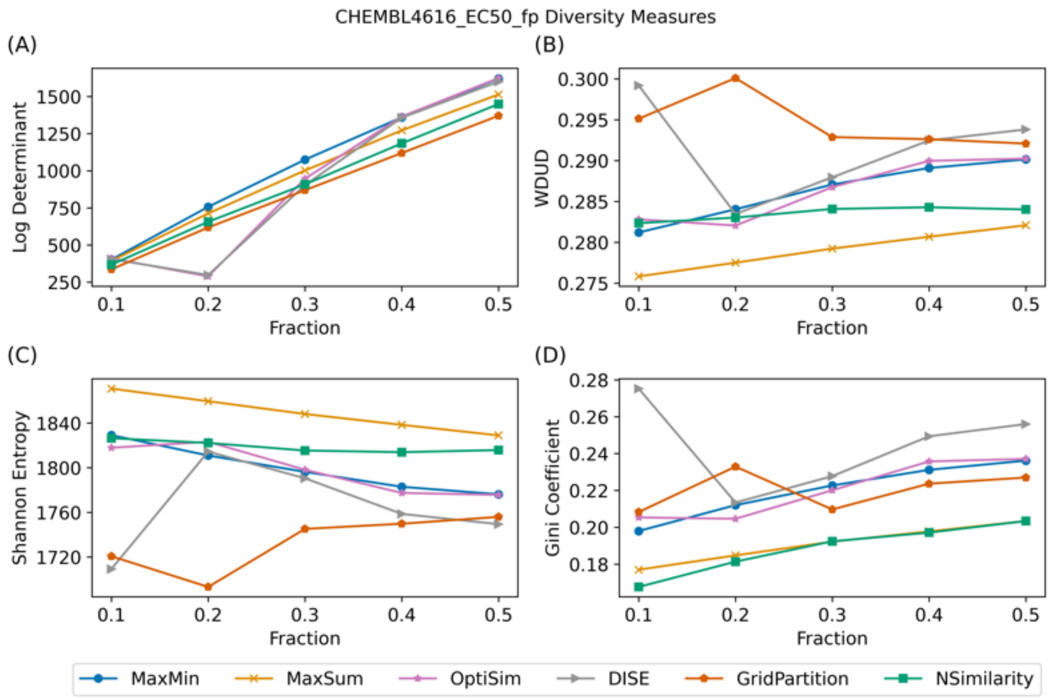

Fig. SI 33 Diversity selection measurements of ChEMBL4616 EC50 subset ($n_{total} = 682$) selection with different selection methods. (A) Log-determinant of the selected subset. (B) Wasserstein distance to uniform distribution (WDUD) of the selected subset. (C) Shannon entropy of the selected subset. (D) Gini coefficient of the selected subset.

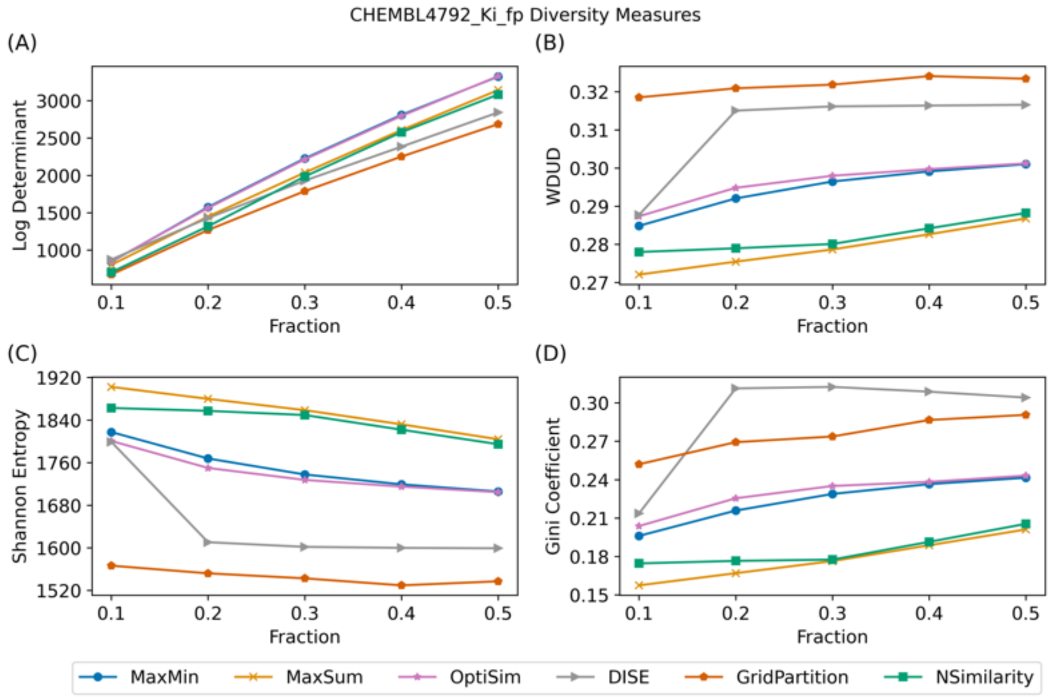

Fig. SI 34 Diversity selection measurements of ChEMBL4792 Ki subset ($n_{total} = 1471$) selection with different selection methods. (A) Log-determinant of the selected subset. (B) Wasserstein distance to uniform distribution (WDUD) of the selected subset. (C) Shannon entropy of the selected subset. (D) Gini coefficient of the selected subset.

References

(1) Mansouri, K.; Grulke, C. M.; Judson, R. S.; Williams, A. J. OPERA models for predicting physicochemical properties and environmental fate endpoints. *Journal of Cheminformatics* **2018**, *10*, 1–19.

(2) Dey, B. K.; Ayers, P. W. A Hamilton–Jacobi type equation for computing minimum potential energy paths. *Molecular Physics* **2006**, *104*, 541–558.

(3) Liu, Y. L.; Burger, S. K.; Dey, B. K.; Sarkar, U.; Janicki, M.; Ayers, P. W. In *Quantum Biochemistry*; Matta, C. F., Ed.; Wiley-VCH: Boston, 2010.
